# On the Science-Policy Bridge: Do Spatial Heat Vulnerability Assessment Studies Influence Policy?

**DOI:** 10.3390/ijerph121013321

**Published:** 2015-10-23

**Authors:** Tanja Wolf, Wen-Ching Chuang, Glenn McGregor

**Affiliations:** 1 Department of Geography, King’s College London, London WC2R 2LS, UK; 2 School of Sustainability, Arizona State University, Tempe, AZ 85281, USA; E-Mail: wen-ching.chuang@asu.edu; 3 Department of Geography, Durham University, Durham DH1 3LE, UK; E-Mail: glenn.mcgregor@durham.ac.uk

**Keywords:** heat stress, vulnerability, mapping, decision support, implementation, awareness, local authorities

## Abstract

Human vulnerability to heat varies at a range of spatial scales, especially within cities where there can be noticeable intra-urban differences in heat risk factors. Mapping and visualizing intra-urban heat vulnerability offers opportunities for presenting information to support decision-making. For example the visualization of the spatial variation of heat vulnerability has the potential to enable local governments to identify hot spots of vulnerability and allocate resources and increase assistance to people in areas of greatest need. Recently there has been a proliferation of heat vulnerability mapping studies, all of which, to varying degrees, justify the process of vulnerability mapping in a policy context. However, to date, there has not been a systematic review of the extent to which the results of vulnerability mapping studies have been applied in decision-making. Accordingly we undertook a comprehensive review of 37 recently published papers that use geospatial techniques for assessing human vulnerability to heat. In addition, we conducted an anonymous survey of the lead authors of the 37 papers in order to establish the level of interaction between the researchers as science information producers and local authorities as information users. Both paper review and author survey results show that heat vulnerability mapping has been used in an attempt to communicate policy recommendations, raise awareness and induce institutional networking and learning, but has not as yet had a substantive influence on policymaking or preventive action.

## 1. Introduction

The direct and indirect health effects of a changing climate, along with an increasing frequency and intensity of extreme weather events such as storms, floods, cold spells and heat waves are considered to be this century’s challenge for society [1,2]. Cities are of particular interest in the context of climate variability and change because this is where potentially a large number of people are exposed to threats from extreme climate events [3]. This is made more germane given current rates of urbanization with just over 50 percent of the world’s population currently living in urban centers. It is estimated that nearly 9% of the world’s population will be living in just 41 megacities by 2030 (UN, 2015). At the same time, the high concentration of people in urban areas, make cities a good place for innovation and social learning. For example initiatives such as the C40 Cities Climate Leadership Group (http://www.c40.org) have resulted in actions that will reduce greenhouse gas emissions and address climate risks and impacts locally and globally, through creating a network of megacities that share knowledge. In relation to this transformative approach to dealing with climate risk and as the paradigm of adaptive management becomes increasingly accepted, it is becoming clear that joint action at the community level, in tandem with top-down interventions from public health agencies are needed to promote resilience to climate change and other stressors [4].

Vulnerability assessments increase the understanding of complex processes and aim at providing decision support for stakeholders and government at a variety of levels. Ideally priorities in adaptation should target areas in greatest need. Spatial assessments of vulnerability are especially appealing to policymakers, as vulnerability maps provide a convenient way to communicate risk related information [5,6]. During the past two decades, the increase in data availability, computational processing and storage capability and the development of geospatial analysis techniques has advanced the possibilities of spatial assessments [7]. Pelling [8], Birkmann and Fuchs [9,10], provide a critical view of both deductive and inductive modeling assuming that vulnerability mapping can induce institutional learning at multiple levels and Hess *et al.* see it is part of a repository of tools that help to improve adaptive management and increase the resilience of local public health systems [11]. Vulnerability mapping has been developed for health risks related to hazards such as earthquakes [12], flood, infectious diseases [13] and others. For example, English *et al.* (2009) reviewed a number of environmental health indicators for multiple hazards associated with climate change (heat stress, flooding, fire, drought, allergens, infectious disease) that included vulnerability indicators among others [14].

Of the aforementioned range of hazards, heat or extreme high temperature events have begun to receive an increasing amount of attention because of their discernible impacts on health and infrastructure. The health impacts of recent heat events such as the 2003 European [15], 2009 Melbourne [16], 2010 Russian [17] and 2014 Japan heatwaves [18] have been considerable, either in terms of number of deaths or those hospitalized. Accordingly, and occasionally at the encouragement of local authorities, researchers have begun to explore the possibility of heat vulnerability mapping as an aid to the development of adaptive management strategies focused on heat as a health problem. Since Smoyer [19] and Wilhelmi [20] called for developing integrated, spatial vulnerability assessments for heat, at least 26 papers on this subject have been published. Theoretically, integrated spatial assessments of vulnerability to heat stress, with the intent to identify hot spots of vulnerability, should assist with indicating where health protection from extreme heat should receive major attention. Reid [21], Maier [22], Harlan [23] and Wolf *et al.* [24] have tested the performance of heat vulnerability indices (HVIs) in different cities and claim, because of the utility in relation to policy development, that they are more than theoretical constructs. Very recently, and therefore not included in this analysis, is Morabito’s [25] mapping heat-related elderly risk index and surface temperature. Bao *et al.* [26] have provided a review of some HVIs, comparing in detail the different input variables.

The question however remains, whether policy makers use the outputs from vulnerability mapping studies. Given this, the goal of this paper is to comprehensively explore the extent to which the published literature on heat vulnerability mapping has been taken up by the policy development community. In relation to this overarching aim, specifically we address the following questions

(1) how has the research concerning heat vulnerability indices (HVI) been conducted, in terms of methods and data used (inductive *versus* deductive)?
(2) what are the limitations and potential problems of current approaches to developing HVIs;
(3) what are the policy recommendations of the research?
(4) are there discernible linkages between HVI research and policy application?
(5) what is the degree of interaction and collaboration between the heat vulnerability research and policy making communities?

By exploring these questions through the critically reflective review and by surveying the authors, our hope is to identify whether current practice facilitates or impedes the inclusion of heat vulnerability (science) mapping in policy making. By analyzing the challenges and limitations in building and applying HVIs we make a contribution to the wider question of bridging the gap between (HVI-) research and policy making. Given the burgeoning number of vulnerability index development and mapping studies and the need for the information generated by such studies to be useable in a policy sense, the aim of this paper is to establish whether there is a gap between research and practice. Accordingly we were interested in posing and answering the question “is there evidence for the application of research findings from vulnerability index and mapping studies to policy development?”

## 2. Methodology

In order to address the above mentioned aim we identified and reviewed “heat vulnerability mapping papers” that had been published up until 15 April 2015; a total of 37 papers were identified. In addition we invited the lead authors of each paper to partake in a short online survey (see Appendix A) composed of 10 core questions designed to elicit information related to vulnerability assessment development and whether the resultant information was incorporated into the formation of any local to regional heat management strategies. In the following sections the methodology for identifying the 37 papers along with the related review criteria and the nature of the online survey questions are described. Paper review results and an analysis of author survey responses is then presented, followed by a discussion of the review and survey results in the context of main question posed by this study.

### 2.1. Paper Identification and Review Criteria

The criteria used in the literature search was informed by previous research undertaken by the authors [24,27,28]. Specifically we used the web resources PubMed.gov (US National Library of Medicine, National Institute of Health), Web of Science and Science Direct to identify heat vulnerability mapping papers. The following keywords were used to search for relevant peer-reviewed articles: Heat, vulnerability to heat, heat waves, and vulnerability mapping. We then screened the search results and kept the papers that applied geospatial techniques. The search was limited to papers published in the English language. Papers were then screened for information about the approach used, policy recommendation given and any indications concerning cooperation with local stakeholders or links between science and action. Information was extracted and summarized in three tables. Based on the extracted information, a qualitative classification of the nature of cooperation between the vulnerability index/mapping producers (“the scientists”) and the decision makers was undertaken; the categories used were “Yes”, “No” and “Vague”. Further, the policy recommendations in these papers were also extracted (see Appendix B).

### 2.2. Author Survey

A survey was set up using an online tool for surveys (eSurv.org). The aims of the survey were to: (a) identify grey literature that provides evidence of the application of the different heat vulnerability indices in local settings; (b) collect examples of good practice of knowledge transfer and cooperation between scientists and local decision makers; (c) understand limitations and obstacles in bridging science and policy. Nine multiple-choice and three open questions (see Appendix A) were posed to the 34 corresponding authors of the papers listed in Table 1, Table 2 and Table 3 (some authors have multiple papers). The questions covered data collection for the development of the heat vulnerability index, the interaction with local authorities, the impact of the work in terms of application of the index and awareness raised, as well as the whether the index was being used to support decision-making. Two questions explored if the authors plan to undertake further research on heat vulnerability or other vulnerability mapping. The consent to publish the survey results was also requested. Authors were invited to partake in the survey in May 2015 and two follow up reminders were subsequently sent to non-responding authors.

## 3. Results

### 3.1. Results of the Literature Review

Based on the search criteria, 37 papers were identified. 21 papers were from the USA and Canada, twelve from Europe and four from Australia. The number of spatial units used for vulnerability mapping varied from 15 (local-government-area scale) to 92,000 (census-block scale). The GIS intrinsically implied possibility to “overlay” risk from hazard (high temperature), exposure to it and population vulnerability is further referred to as the “Crichton’s Risk Triangle” approach [25,29,30]. A series of studies reduce the dimensionality of data of several input variables. The studies identified by the literature search are displayed in tables 1, 2 and 3 according to the approach used to construct the vulnerability index, namely: (1) “*a-priori*”/inductive index without testing with empirical health data (11 studies); (2) “*a-priori*”/inductive index and with testing with health data (6 studies, two are the testing part of studies from Table 1); and (3) a “bottom-up” approach to mapping health outcomes and subsequently exploring vulnerability factors (9 studies). These studies analyze health data to derive information about spatial vulnerability patterns from empirical data. We did not observe any HVI studies that adopt a deductive approach, which utilizes available theories or frameworks to derive an HVI—this may be because there is lack of a strong theory or framework for developing vulnerability indices [31], and vulnerability being a “place-based” concept which makes it rather context specific.

Table 1 summarizes the details of 18 studies that use census data and methods of dimensionality reduction, such as Principal Components (PCA) or Factor Analysis, to produce an “*a-priori*” vulnerability index that is then mapped. Such an approach could be considered as a “top–down” approach. Tate refers to this type of research method as “inductive” [32], as it uses variables that directly or in a proxy sense represent heat risk factors, to construct vulnerability indices. Reid, for example, performs a Factor Analysis of ten variables (demographics, prevalence of air condition, vegetation cover from satellite images and diabetes rate) and combines the resulting components into a vulnerability index for urban areas in the US [33]. Some studies also include projections of climate and population and thus try to estimate future vulnerability [34,35]. In some cases, the heat/temperature hazard is mapped separately [28], in others it is integrated into the index [36].

Table 2 summarizes details of seven studies that apply the “top down”- approach as outlined above for Table 1 but further use spatial health data to test the performance of the index or elements of it. Wolf *et al.* [24] and Reid et al [21] in fact describe the testing/validation of vulnerability indices based on PCA or Factor Analysis presented in separate papers. Also risk-mapping studies [37] using health data are included here.

Table 3 summarizes details of the remaining twelve studies with a “bottom-up” approach: They all use health data to weight components in a HVI or directly map adverse health outcomes (e.g., excess heat deaths or heat related illness) and then identify characteristics of the vulnerable populations using an array of statistical methods. The heterogeneous methods here range from General Linear and Mixed Models applied to different factors for heat mortality and heat distress calls [38], Principal Component Analysis with inclusion of mortality data into the analysis rather than for validation [39,40], identification of areas with excess mortality and subsequent identification of risk factors through principal component regression [41], mapping of heat related mortality rate ratios and evaluation of spatial association between variables that describe neighborhood-scale characteristics and excess deaths amongst the elderly [42], vulnerability and exposure modeling with standard linear regression relating temperature to morbidity and mortality indicators [43], a forward selection algorithm based on Bayesian information criterion [44] and a series of hierarchical Bayesian models to examine associations between temperature and morbidity [45].

**Table 1:** Papers on “*a-priori*”/inductive heat vulnerability mapping.

Reference	Study Area	Spatial Unit	Approach	Cooperation with Decision Makers?
Vescovi *et al.* 2005 [34]	Southern Quebec, Canada	Census subdivision (similar to municipalities)	Synthesis and overlay of present and future climate hazard and four social vulnerability sub indices.	YES: Research is intended to feed into decision making: “This study gives preliminary input to the Quebec public health decision-makers who intend to develop a spatially explicit on-line analytical processing tool using Web-GIS technology to identify areas vulnerable to climate change.” (p. 77)
Lindley *et al.* 2006 [46]	Manchester, the United Kingdom	Census block	Mapping of current and future temperature, land use through aerial photography, indicators representing vulnerable groups (current and population projections, projection of income disparity).	YES: Reference to a joint workshop is made: “.... these were factors raised as important in a recent evaluation workshop held with local advisors from a range of government and non-governmental organizations” (p. 565).
Reid *et al.* 2009 [33]	Metropolitan statistical areas, USA	Census tract (with minimum 1000 people)	Factor analysis of ten variables (demographics, prevalence of air condition use, vegetation cover from satellite images and diabetes prevalence); national coverage of urban areas; evaluation with health data in separate paper (see Table 2).	NO: Not specifically mentioned here, rather pure research.
Rinner *et al.* 2009 [47]	Toronto, Canada	Census tract, dissemination area, city neighborhood	Composite indices from satellite thermal image and ordered weighted averaging of multi criteria operators (general population and targeting seniors).	YES: Clear link to the City of Toronto, Toronto Public Health, Medical Officer of Health in Greater Toronto Area who has requested this information to support decision-making processes. The SIMMER project and an evaluation report [48] are linked to this initiative.
Kershaw and Millward 2012 [49]	Toronto, Canada	120 m pixels	Exposure only: prediction and mapping of humidex degree hours, integrating apparent temperature intensity and duration.	NO: Although the research is part of the above mentioned SIMMER project, this work is about methods to model and assess the exposure to heat and cooperation with decision makers is not relevant.
Chow *et al.* 2012 [50]	Metropolitan Phoenix area, AZ, USA	Census tract	Comparison of vulnerability in 1990 and 2000 based on a composite index of vulnerability, equal weight of physical exposure to heat and four socioeconomic measures.	NO: Paper refers to longstanding vulnerability research in Phoenix, but no indication of links to policy making or action is highlighted in the paper.
Wolf *et al.* 2013a [28]	London, United Kingdom	4765 Census district	Principal components analysis of nine proxy measures reveal four components; weighted according to the variance they explain these are summed to form the HVI. Evaluation with health data in separate paper (see Table 2).	VAGUE: Cooperation with Greater London Authority (GLA) and data providers is acknowledged. Further research on heat in London is ongoing but direct link to this work is not obvious.
Depietri *et al.* 2014 [36]	Cologne area, Germany	85 districts	Vulnerability to heat waves is calculated by normalizing and aggregating the composite indicators: socio-economic data, remote sensing data in the form of thermal infrared imagery, land-use and land-cover classification maps, and a map of the forest cover.	YES: This works seems to be imbedded into local level. Stakeholders’ interviews were carried out to investigate the perception of local authorities regarding the capacity to mitigate the impacts of heat waves (p. 102).
El-Zein and Tonmoy 2015 [51]	Sydney, Australia	15 local government areas	Comparison of rankings generated by the outranking approach to those yielded by additive and multiplicative aggregations. Vulnerability to heat was represented by a set of 6 indicators representing exposure, 4 indicators for sensitivity and 12 indicators for adaptive capacity.	NO: This paper defends a specific method to assess vulnerability to heat stress and does not give further indication on links to action.
Buscail *et al.* 2012 [29]	Rennes, France	92 census block groups	Hazard and vulnerability indices were combined to deliver a heat-wave health risk index.	NO: No particularly close links to policy makers.
Aubrecht and Özceylan 2013 [52]	US National Capital Region (Washington D.C., and the surrounding metropolitan area consisting of parts of the U.S. states of Maryland, Virginia, and West Virginia).	Census block level (22 counties, 3500 census block groups 92,000 census blocks)	Score of the heat stress risk index (HSRI) as multiplication of two equally weighted risk components: number of heat wave days and vulnerability defined by selected population and land cover characteristics.	VAGUE: “Last but not least, the developed risk identification and mapping approach will be promoted in the relevant public health communities, aiming at providing decision support for municipal and local heat response planning.” (p. 75).
Tomlinson 2011 [30]	Birmingham, United Kingdom	641 Lower Layer Super Output Area” (LSOA)	Spatial coincidence of Hazard Layer (urban heat island) and four vulnerability /filtered exposure layers build the Risk Layer.	VAGUE: “It is anticipated that the results of this work will be incorporated into a spatial decision support tool where the weightings can be altered according to specific user requirements”(p. 4).
Van den Hoeven and Wandel 2015 [53]	Amsterdam, The Netherlands	Different spatial resolutions for different data	Simple mapping of elements contributing to vulnerability such as surface temperature in the city, the spatial distribution of its population and workforce, the energy efficiency of the buildings and the quality of life in the neighbourhoods.	YES: Research was developed with policy makers in the project Amsterwarn and conducted in the framework of the Climate Proof Cities pro- gramme that works for strengthening the adaptive capacity and reducing the vulnerability of the urban system against climate change and to develop strategies and policy instruments for adapting our cities and buildings.
Dugord *et al.* 2014 [54]	Berlin, Germany	Small-scale building block level	Mapping of potential heat-stress risk across the city by aggregation of values (0 to 4 according to 95th to 85th percentile of distribution of hazard (urban air temperatures) and vulnerability (population density, concentration of vulnerable inhabitants (population density, percentage of vulnerable inhabitants due to high or low age)	NO: Research project, giving recommendations for urban planning.
Merbitz *et al.* 2012 [55]	Aachen, Germany	Multi-scalar analyses which include points and buffer circles with 200 m, 400 m and 800 m radius.	Identification of hot spots with high health risks for distinct groups of urban population, measurement campaigns were carried out, capturing the spatial distribution of temperature and PM concentrations in the City of Aachen, Germany.	VAGUE: “The study is embedded in the project City2020+ which is part of the interdisciplinary Project House HumTec (Human Technology Center)”, which may enhance application of research findings in the future.
Oven *et al.* 2012 [56]	United Kingdom	Different grids	Spatial distribution of projected future hazard to heat (and cold and flooding) as well as future shares of older populations as the more vulnerable are visually inspected.	NO: but envisaged for the next stages of the project. It is planned to assess the potential to apply geographical mapping as part of the consultation and planning process at local level. Stakeholders in local communities, and at national and international levels, will be consulted with the aim of determining how effectively this kind of information (combined with finer scale maps at the local level) can support resilience planning processes (p. 23).
Keramitsoglou *et al.* 2013 [57]	Athens, Greece	1km grid, census blocks	Fuzzy logic used to create monthly heat wave risk maps, an integration of modeled heat wave hazard and geospatial information on the population vulnerability to heat waves calculated from two census variables (population density and percentage of non-proper dwellings).	NO: Research only. It provids decision support and a repeatable, low-cost method for identifying vulnerability maps. Testing of the index is considered desirable but not possible yet. “Ultimate validation exercise is to compare the output hazard and risk maps against spatially distributed morbidity and mortality data; at the stage of publication and to the knowledge of the authors, such dataset is not available for Athens (p. 8253)”.
Norton *et al.* s2015 [58]	City of Port Phillips, Australia	228 statistical areas	Overlay of exposure (daytime and night time temperature), vulnerability (population aged over 65 and below 5 years old, socioeconomic disadvantage) and areas of population behavioural exposure (public places).	ES: The assessment of priority areas for mitigation in form of urban green infrastructure was undertaken with the support of the City of Port Phillip and with local council representatives from across Melbourne in a workshop.

**Table 2:** Papers on “*a-priori*”/inductive heat vulnerability mapping using health data to test the index.

Reference	Study Area	Spatial Unit	Approach, Evaluation of HVI with Health Data?	Cooperation with Decision Makers?
Reid *et al.* 2012 [21]	California, New Mexico, Washington, Oregon and Massachusetts, USA.	Zip-code area	Testing if HVI (Reid 2009) is indicator for heat related health outcomes.	YES: This study is the result of a data linkage project within the Centers for Disease Control and Prevention’s (CDC) National Environmental Public Health Tracking (EPHT) Network in which researchers at the University of California-Berkeley (UCB) collaborated with public health professionals from EPHT programs in several states.
Harlan *et al.* 2013 [23]	Maricopa County, (in Phoenix Metropolitan Area) AZ, USA	Census-block group	HVI sums eight aggregated neighborhood population characteristics, including prevalence of air conditioning (AC), and amount of vegetation cover PCA; evaluation of HVI with heat related mortality including evaluation of the role of surface temperature.	VAGUE: Cooperation with Maricopa County Department of Public Health; Arizona State University’s Center for Health Information Research; further application not clear.
Wolf *et al.* 2013b [24]	London, United Kingdom	4765 Census districts (Lower layer Super Output Area (LSOA)	Three approaches to test the HVI presented above are explored using mortality data and ambulance callout data.	VAGUE: Cooperation with GLA and data providers is acknowledged.
Maier *et al.* 2014 [22]	Georgia, USA	County level (159 counties)	HVI built from factors of PCA from eight demographic, heath, and land use/land cover data variables; testing with all cause mortality.	NO: Work appears to be linked well with similar research in the US, but not with policy.
Chuang *et al.* 2015 [27]	Phoenix, Arizona, USA	362 census tracts	Using factor scores from a factor analysis as independent variables, and heat hospitalizations as dependent variables in a multinomial logistic regression model, the paper evaluated the accuracy of the index in a local context.	NO: Policy links not given.
Crider 2014 [37]	Alabama, USA	16 metropolitan statistical areas (MSAs)	A weighted occupation-based metabolic equivalent (MET) index was created. The correlation between current MET-weighted employment rates or obesity rates and 2012 heat related Illness (HRI) report rates in Alabama were then determined.	NO: The author is affiliated to a local School of Public Health, the use of the elaborated information is not described and it seems to remain rather research than practice at the moment.
Houghton *et al.* 2012 [59]	Austin, Travis county, Texas, USA	Census-block group	A non-weighted index of vulnerability was created for extreme heat (and flooding) using PCA. Comparison with health data to identify possible hotspot clusters of populations with both high vulnerability and high mortality rates.	VAGUE: In the context of this work, not only heat vulnerability maps are developed, also other flooding and climate-relevant policies (such as Municipal tree planting) are integrated in a webtool, the Geospatial Emergency Management Support System (GEMSS), a geospatial clearinghouse and data services network (p. 43).

**Table 3:** Papers on mapping health outcomes and exploring vulnerability factors.

Reference	Study Area	Spatial Unit	Approach	Cooperation with Decision Makers?
Uejio *et al.* 2011 [38]	Philadelphia, PA; metropolitan Phoenix, AZ, USA	Census-block groups	Comparison of relative importance of different factors for heat mortality/heat distress calls in two cities, mapping of Observed and fitted Generalized Linear and Mixed Model.	NO: No policy link mentioned.
Johnson *et al.* 2012 [39]	Chicago, IL, USA	Census-block group	25 indicators of extreme heat-health risk are combined into an applied index utilizing a principal components analysis. Here mortality data is included in the index.	NO: Many practical recommendations and research suggestions, but link to policy is not clear from the paper.
Hondula *et al.* 2012 [41,60]	Philadelphia County, PA, USA [41]; 7 U.S. cities [60]	Zip code tabulation area	Areas with mortality exceedances were identified using randomization test. The environmental, demographic, and social factors associated with high-risk areas were identified via principal components regression.	Vague: The department of health in Pennsylvania provided mortality data. The authors are adopting this approach for other United States cities in different climate zones to determine if certain factors are consistently associated with elevated risk during heat waves.
Boumans *et al.* 2014 [43]	Travis County, Texas, USA	696 Travis County census/watershed units	Vulnerability and exposure modeling include standard linear regression equations relating temperature to mortality and morbidity indicators	YES: Close link of this model building project to policy as the work is result of a ‘‘participatory modeling workshop’’ to develop a tool for decision-makers in estimating climate change effects on human health and health—environment interactions, convened in December 2010 by a consortium of EPA, Centers for Disease Control, and state and local health officials in Austin, Texas.
Heaton *et al.* 2014 [44]	Houston, Texas, USA	Census blocks	A forward selection algorithm based on Bayesian information criterion (BIC) is used to identify which of the exposure, sensitivity, and adaptive capacity variables are explanatory of non-accidental mortality.	NO: No clear link to policy application of the results is made, this work rather stimulates further research.
Loughnan *et al.* 2012 [40]	Melbourne, Australia	Postal area	Eight environmental, health, and demographic variables were summed up to a spatial heat vulnerability index by weighting the variables according to a value from a stepwise multiple regression between the variables and the adverse health outcome (anomaly in daily emergency admissions and mortality).	VAGUE: This work is related to a project with similar approach in all capital Australian cities (Brisbane; Canberra; Darwin; Hobart; Melbourne; Perth; Adelaide; Sydney) [61]
Klein Rosenthal *et al.* 2014 [42]	New York City, USA	59 community districts and 42 New York City United Hospital Fund (UHF) neighborhoods	Mapping of mortality rate ratios for seniors age 65 and older (hot days compared to all summer days and evaluation of spatial association between independent variables that describe neighborhood-scale characteristics and senior citizens’ rates of excess deaths during heat events.	YES: Close interaction with people at New York City Department of Health and Mental Hygiene.
Johnson and Wilson 2012 [62]	Philadelphia, USA	Block group level	Utilizing variables from an exploratory analysis (standard deviational ellipse) a multiple linear regression model using UHI intensity and vulnerable population characteristics is developed to predict EHE mortality.	NO: Funding from Centers for Disease Control and Prevention, no indication of further collaboration with stakeholders.
Hondula 2014 [45]	Brisbane, Australia	158 Statistical Local Areas	Series of hierarchical Bayesian models to examine city-wide and intra-city associations between temperature and morbidity using a 2007–2011 time series of geographically-referenced hospital admissions data.	NO: The cooperation with decision makers seems to be less relevant in this research.
Schuster *et al.* 2014 [63]	Berlin, Germany	397 Planning areas	Mapping of age-standardized mortality rates by calculating the relative heat mortality risk ratio for months with and without severe heat waves. Local indicators of spatial association were used to locate spatial clusters.	NO: The cooperation with decision makers seems to be less relevant in this research.
Kovach *et al.* 2015 [64]	Rural and urban NC, USA	ZIP code level	Spatial regression of 11 potential demographic, socioeconomic, and land cover risk factors to determine whether they have a statistically significant association with rates of HRI.	NO: research only.
Hattis *et al.* 2012 [65]	Massachusetts, USA	29 municipality groups	Analysis of the spatial distribution of heat-related mortality in relation to both urbanization and relevant socio-demographic variables.	NO: Research about factors determining heat-related mortality rather than vulnerability mapping.

In 15 out of the 37 papers identified geo-referenced health outcome data is used. The type of health outcome data used in the studies listed in Table 2 and Table 3 varies. There are, for example, different ways to identify “heat related” mortality: in some studies mortality includes “all causes”, in other studies some specific causes of death (e.g., “external causes”) are excluded. The same applies to morbidity measures such as (all or selected) hospital admissions and (all or selected) ambulance calls.

Indications relating to cooperation with local stakeholders or links between science and action were extracted and are summarized in Table 1, Table 2 and Table 3 and classified into “Yes”, “No” and “Vague”. This categorization reveals that most studies in group 1 (inductive/top down approach without health data) (6 out of 18 papers) give an indication about concrete cooperation with decision makers, whereas this can be confirmed for only one study (out of 7) in group 2 (inductive/top down approach with health data testing). Group 3 (bottom-up approach with use of health data) records 2 “Yes” (out of 12). For details see Table 4. The papers’ information about policy recommendations is summarized in Table A1, Table A2 and Table A3 in Appendix B.

**Table 4:** Cooperation with decision makers, determined by the review of literature.

Group	YES	VAGUE	NO	*Total*
Top–down without health data (Table 1)	6	4	8	*18*
Top–down with health data (Table 2)	1	3	3	*7*
Bottom-up with health data (Table 3)	2	2	8	*12*
*Total*	*9*	*9*	*19*	*37*

### 3.2. Results of the Survey

Authors were invited to partake in the survey in May 2015 and two follow up reminders were subsequently sent to non-responding authors; 21 out of 34 authors completed the survey by closure of the survey in July 2015 (61% response rate). The replies were as follows:

- *Data collection:* Most of the data were available for free (76%), a minor fee of less than US$100 or equivalent was charged to some (10%) and 14% paid a higher fee. Although not specified by the respondents, some of the high costs may be related to the acquisition of satellite images Data were available online for 29% of the respondents. Local authorities were supportive in data collection (48%) or helpful after considerable follow up by researchers (19%).
- *Interaction with local authorities:* The levels of interaction with local authorities varied. 86% reported that there was interaction with local authorities at different levels. 24% report much interaction (oral presentation/discussion at conferences, meetings or workshops, joint publications, email and phone), 38% “some” interaction and 24% “some but not much” interaction. 71% report that local officials commented on the vulnerability index, 29% did not know or did not get feedback. The overall tenor of the discussions and comments received by the survey respondents was considered fruitful and constructive. Only 14% of the respondents reported that there was no interaction. This probably applies to those studies with a pure focus on research where the exploration of the vulnerability assessment was the primary goal.
- *Use of the analysis:* Overall, respondents were positive about their index being applied. 71% think—to low, mid and high degree—that the respective vulnerability index is or will be used to support decisions on where to take action. Further, more than half of the respondents think that the results of the work are or will be applied in the local context: 14% thought the research results have already had significant influence in the local context and 43% saw or envisaged some local application. But there are also skeptics: 14% of respondents thought that the degree of application was very limited. 19% of respondents do think that results are not being applied in the local context and 10% of respondents do not know. 29% of respondents replied “I don’t know” to the question “do you think the index is or will be used to support decisions on where to take action”.
- *Awareness raised:* 90% of the respondents think that the respective work has increased awareness among the authorities and/or in the public to a low (38%), mid (33%) or high (19%) degree.
- *Risk communication:* 76% stated that the work has been used to communicate risks. In 40% the work was used by researchers to communicate risks to local agencies and/or experts or to the general public (30%). Only in 6% the local authorities were considered active in risk communication to the general public using the scientific work.
- *Further research*: 76% of the replying authors are planning to undertake further work on the topic of vulnerability to heat in the same or another urban area, 5% exclude this and 19% do not know. Consideration given to building a vulnerability index for other hazards is rather scarce, half of the respondents replied “no” or “do not know”. Some also claim that similar criteria (social cohesion) define vulnerability to heat as well as other hazards. Others have done, know about or envisage vulnerability mapping for flooding and for several other hazards (severe storms, tsunamis, droughts, wild fires, disease vectors, earthquakes, land-slides environmental refugees, food shortages).

## 4. Discussion

This literature review has shown that there have been numerous attempts to assess and map vulnerability to heat stress in urban areas. Among the 37 papers reviewed, almost half adopted a similar set of methods and variables that were established by a few foundation studies, such as Cutter [66]. This indicates that Cutter’s inductive approach to developing a SoVI (Social Vulnerability Index) for environmental hazards in general, significantly influenced the majority of subsequent heat vulnerability work. The use of ready and often freely available (census) data and by now relatively accessible computation methods (PCA/factor analysis) has provided the opportunity to apply a “Cutter-type” approach to a variety of different urban areas. However, Tate [32] has indicated that the SoVI has limitations in measuring actual vulnerability; these weaknesses apply to some HVI as well. Indicator selection according to local availability, scale of analysis, measurement error, data transformation, normalization, and weighting as well as aggregation are all possible sources of index inaccuracy. In addition, the contextual effects from the complex interactions of a place’s unique socio-ecological systems could also limit the predictability of generic vulnerability indicators [55]. Further, as Romero-Lankao [67] concludes in her meta-analysis, researchers often make rather subjective modeling decisions with little or no stated justification in urban heat vulnerability index studies. While she argues that subjectivity is not inherently a bad thing and inevitable to some extent, she underlines that the effects of subjective choices on the output index need to be assessed before applying and using it to prioritize action. Similarly, Tate [32] claims that uncertainty and sensitivity analysis is often missing in such research. This is largely borne out by the review of studies presented in this paper.

While uncertainty originating from model development procedures is acknowledged in some papers (e.g., [68,69]), the implications that uncertainties associated with vulnerability assessment hold for policy development and decision making are not addressed explicitly. In fact, the way in which many of the papers present the science in relation to policy fits the linear communication model [70] such that information is provided with the expectation that there will be uptake by a relatively amorphous policy community. Some researchers have referred to this as the “loading dock” approach [71] to the provision of scientific information for policy development and have expressed the inappropriateness of this method, especially in the case of communicating scientific content that may possess elements of uncertainty [72]. In a similar vein, Knaggard [73] highlights the issues associated with evidence-based decision-making in the context of a paradigm of rationality. Despite the best intentions of some of the studies reviewed here, the fact that uncertainty in relation to policy and decision making is not addressed unambiguously raises the possible spectre of unusable science or non-actionable knowledge [74,75]. Although this situation is regrettable it is not irretrievable as the opportunity exists to adopt some imaginative approaches to assessing or simulating the effects of uncertainty on policy development in future studies that build on those reviewed here. For example van Pelt *et al.* [72] discuss the utility of intermediaries or boundary objects in acknowledging and communicating uncertainty and Head [76] outlines strategies for addressing the challenges arising from so called “wicked problems”, a category of problem in which the impacts of heat on society comfortably sits. Dany *et al.* [77] and Berkhout *et al.* [78] both emphasize the critical role that stakeholder opinions play in how research and policy can develop in a mutually beneficial way and Reed *et al.* [79] suggest a number of principles that should be followed for effective practice of knowledge exchange.

Given that many of the heat vulnerability indices reviewed here have been developed in isolation of local knowledge, not only about micro-climates but also known socially determined hotspots of vulnerability, the suggestion by Kniveton *et al.* [80] that integrating local and scientific knowledge can be beneficial for addressing uncertainty, is particularly pertinent. Millner *et al.* [81] also describe how expert elicitation can assist with gauging the impact of uncertainty on decision making.

While there is agreement on the awareness raised (only 10% are not sure about this), several respondents claim that the application of the developed index at the local level is limited. Only 14% think that the research results have had a significant influence in the local context. When it comes to the use of the index to support decisions, merely 5% of the respondents clearly stated “yes, to a high degree”; 29% replied “I don’t know” and 43% (24%) replied yes, to a mid (low) degree. This shows that the mapping and index development does seem to have a positive effect in awareness raising, but less so in triggering action or supporting decisions. Alternatively, the researchers are simply not informed to what extent their scientific results are used for decision support.

The inherent subjectivity and uncertainty of the index development methods, although known but not explicitly expressed by the index developers, could be the reason for limited uptake. Accordingly Tate underlines that the addition of uncertainty analysis to the index construction process is therefore an important step toward improving the quality of the next generation of social vulnerability indexes. Epistemic uncertainty is associated with all vulnerability models. The lack of its assessment and portrayal does not deny its existence [32]. Clearly a challenge for the vulnerability index development community, who want their science to be applied in policy making, is how to quantify uncertainties and effectively communicate these. This remains an area to be explored.

Along these lines Romero-Lankao [67] points to another weakness of vulnerabiltiy mapping science when she writes: “equally fundamental dimensions and determinants of vulnerability are ignored just because of lack of data and (*index development*) omits any attempt to gain ethnographic knowledge of behavioural norms, social networks and risk perceptions that are equally relevant to understanding urban vulnerability.” Klinenberg [82] has shown this in his “social autopsy” for Chicago as well. Social cohesion as a protective factor is something that appears even more difficult or impossible to “measure” as a determining component of vulnerability. New perspectives or approaches, such as applying qualitative methods that capture for example, measures of social cohesion, may improve the performance and acceptability of vulnerability indices. Social cohesion could even be of relevance when victims of heatwaves do not belong to the classic risk groups (single, frail elderly) as Duneier [83] showed for Chicago.

Hinkel [31] has confronted the scientific limitations of vulnerability indicators and the issues they profess to address. He concludes that vulnerability indicators (and indices) are only good for identifying vulnerable people, communities or regions. Other common expectations regarding indicators such as identifying mitigation targets (and adaptation actions), raising awareness, allocating adaptation funds, monitoring adaptation policy and conducting scientific research he suggested cannot be fulfilled. This means that the studies identified here seem to best serve purposes (raising awareness, conducting research, identifying adaptation action) that they were not originally intended to serve. Hinkel [31] points to the problems intrinsic to risk communication: accordingly vulnerability indicators (and indices) are not the right means to raise awareness of climate change.

## 5. Conclusions

Heat vulnerability index development and associated risk mapping has developed significantly during the past decade. This is a result of the recognition that levels of vulnerability to heat vary within the population and that certain combinations of a range of risk factors may conspire at the individual to community level to increase the level of vulnerability to heat during extreme temperature events. As for other natural hazards, the visual presentation of vulnerability using coloured maps is experienced and seen as an effective communication device to raise awareness and as a possible input into policy and decision making. The increasing availability of high resolution spatial data at the intra-urban scale and advances in GIS technology have created the possibility for the natural hazards and social determinants of risk communities to create information that will assist with protecting the health of the vulnerable. The hypothesis that information at higher spatial resolution is better absorbed for protective action warrants further testing. However this normative view, based around potentialities and aspirations must be tempered by a positivist view of the current situation. The analysis presented in this paper has attempted to do this.

Although a considerable effort has been invested in heat vulnerability mapping research, the analysis presented here, based on a review of available literature and a survey of vulnerability index mapping paper authors, demonstrates the persistence of an unambiguous gap between vulnerability mapping science and policy. At the heart of this issue is a lack of understanding of how to mainstream this type of science into decision making, notwithstanding of course the issues that something as complex as vulnerability can be captured in a single index and that methodological issues related to vulnerability index construction need to be resolved before proceeding to policy development. Other substantive issues highlighted by this analysis include how effective or accurate (in terms of predicting impacts or outcomes) does a vulnerability index need to be in order to be “reliable”? The answer to this question not only lies with improving the science of vulnerability index based predictions of health responses but rests with the ethics of what is a socially acceptable level of vulnerability assessment inaccuracy. Allied with this is the issue of uncertainty. In relation to this, our analysis of vulnerability mapping papers and author survey results reveal that not only is there an inherent uncertainty associated with the indices on which vulnerability mapping is based, but a critical analysis of how uncertainty may cascade through the decision making and policy development process is lacking in the heat vulnerability mapping literature. This may relate to the nature of interaction that the vulnerability index development community has with policy developers in local authorities, in that it is one-way in the direction of end users, as opposed to a truly reciprocal interaction, which may assist with improving the understanding and communication of uncertainty.

While the results of our analysis point to a number of commonalities within the vulnerability index and mapping literature that may militate against mainstreaming of research outcomes into policy development, we are hopeful that the gap between science and policy will lessen. This will be achieved by both science and policy makers working within a co-production of knowledge paradigm and developing a greater mutual understanding of the barriers, constraints and limitation faced by both communities in terms of what they are striving for, such as the achievement of social justice and the reduction of inequalities in the context of the societal impacts of extreme heat events. In addition to this paper, to gather researchers active in this field to develop common guidelines could be a way forward.

Lastly, it should be acknowledged that this being an emerging field of research with more studies appearing during finalization of the manuscript, this review might not have captured all the published literature on recent heat vulnerability mapping. Further, 39% of the contacted authors did not reply to the questionnaire. Accordingly the findings from the analysis presented here should be treated as only indicative of current heat vulnerability mapping activity, application and use of the research results. To what extent these findings for heat vulnerability mapping may be applicable, or not, to other hazards, is another interesting question to be explored.

## Appendix

### A. Survey Questions

1. For the vulnerability paper(s) you have published, did you perceive the local authorities to be supportive in making data available?
  ○ Yes, local authorities provided some support after considerable following up on my part.
  ○ Yes, local authorities were supportive and helped a lot to get necessary data.
  ○ No. Most data was publicly available online, no support from local authorities was needed.
  ○ No. I did not perceive the local authorities as supportive at all.
  ○ I don’t know.
2. Did you pay a fee for the data?
  ○ No, data was freely available.
  ○ Yes, I paid a very minor fee (less than USD $100 or equivalent).
  ○ Yes, I was charged more than USD $100 or equivalent.
  ○ I don’t know.
3. Did you have a chance to discuss and develop your work and its results with representatives of local authorities?
  ○ Yes, but not much interaction.
  ○ Yes, with some interaction.
  ○ Yes, with much interaction.
  ○ No.
  ○ I don’t know.

If you answer “Yes” above, in what form do you interact with local authorities? (ex. Oral presentation, report, *etc.*)

4. Did representatives/local officials comment on your vulnerability index?
  ○ Yes, but not much.
  ○ Yes, to some degree.
  ○ No.
  ○ I don’t know.
5. Do you think that the results of your work are or will be applied in the local context (ex. municipality)?
  ○ Yes, but I think that the degree of application is very limited.
  ○ Yes, I think results (will) have some local application.
  ○ Yes, I think my research results have had significant influence in the local context.
  ○ No, I think that the results are not being applied in the local context.
  ○ I don’t know

Can you give examples or link to publications when your results have been applied, or explain what limitations are?

6. Do you think that your work has increased awareness among the authorities and/or in the public?
  ○ Yes (low degree).
  ○ Yes (mid degree).
  ○ Yes (high degree)
  ○ No.
  ○ I don’t know
7. Was your work (on mapping vulnerability to heat) used to communicate risk (by any means of risk communication) to, or by local authorities and/or policy makers? (You can select multiple answers)
  ○ Yes, I (or my colleagues) use the work to communicate risks to local agencies and/or experts.
  ○ Yes, I (or my colleagues) use the work to communicate risks to general public.
  ○ Yes, local authorities and/or policy makers used (cited) to communicate risk to the general public.
  ○ No.
  ○ I don’t know.
8. Do you think that your vulnerability index is used or will be used to support decisions on where to take action?
  ○ Yes (low degree).
  ○ Yes (mid degree).
  ○ Yes (high degree).
  ○ No.
  ○ I don’t know.
9. Are you planning further work on the same topic (vulnerability to heat) in the same or another urban area?
  ○ Yes.
  ○ No.
  ○ I don’t know.
10. Are you considering building a vulnerability index for other hazards? If yes, for what hazard and in what area?

### B. Policy Recommendations

**Table A1:** Policy recommendation from “*a-priori*”/inductive heat vulnerability mapping.

Reference	Policy Recommendation
Vescovi *et al.* 2005 [34]	“The most important aspect of our results is the geographical designation of specific zones where people are expected to be at risk in a warmer climate. Specific measures concerning mainly the elderly should be put in place for these regions so that relief can be provided immediately in the event of a heat wave”. (p. 77)
Lindley *et al.* 2006 [46]	“…the method also provides a mechanism through which areas suitable for further neighbourhood scale assessment and potential adaptation strategies can be determined. An analysis of the nature of hazards and vulnerabilities within cities and other urban areas is clearly a useful basis for tailoring planning and design strategies to the specific needs of the affected community”. (p. 565)
Reid *et al.* 2009 [33]	“With further validation at the local scale and evaluation with health outcome data, our methodology and results can help target resources for intervention”. (p. 1735)
Rinner *et al.* 2009 [47]	The recommendations include creating multiple representations of vulnerability indicators, indices and hot spots in order to avoid issues resulting from geographic aggregation and scale effects, variable selection, and the input parameters of cluster analysis and multi-criteria methods.
Kershaw and Millward 2012 [49]	Our results highlight the value to public health organizations of *in situ* meteorological data when evaluating potential vulnerability during extreme heat events. (p. 7340)
Chow *et al.* 2011 [50]	“Anticipate increased heat-related emergency dispatches calls during heat wave events and tailor effective measures for them (e.g., more Spanish-speaking responders or specialized elderly medical aid centers). Policies to improve social cohesion and integration within neighborhoods via widespread dissemination of heat-stress mitigation information in different languages”. (p. 15)
Wolf *et al.* 2013a [28]	“…the index presented here needs to be tested as a reliable *a priori* predictor of health outcomes such as mortality or ambulance call out. This will be the focus of future work”. (p. 67)
Depietri *et al.* 2014 [36]	“Our analysis showed that, while the higher vulnerability of the population of Cologne to heat waves is concentrated in the city center, policies that aim to tackling it should also take into account the connections and interactions between the city center, the surrounding districts and its hinterland, reducing the susceptibility of lower status social groups and enhancing ecosystem management”. (pp. 115–116)
El-Zein and Tonmoy 2015 [51]	“outranking procedures, previously only applied to decision-making problems, can be used for vulnerability assessment and may provide a better approach for teasing out policy-relevant information from uncertain vulnerability data. ” (p. 216)
Buscail *et al.* 2012 [29]	“We recommend, however, using the health risk index together with hazard and vulnerability indices to implement tailored programs because exposure to heat and vulnerability do not require the same prevention strategies”. (p. 8)
Aubrecht and Özceylan 2013 [52]	“Applying a very granular approach at a high level of spatial detail enables the detection of hotspot areas within cities. (…) It can therefore provide valuable decision support in directing risk mitigation measures which in a heat stress context particularly implies increasing the local communities’ adaptive capacity”. (p. 74)
Tomlinson 2011 [30]	“This work offers the foundations for a spatial decision support tool that could be linked to climate change and projection models in order to consider climate change adaptation with a focus on heat health risks. Indeed, such data is potentially of great use to local authorities and health agencies when deciding on targeted campaigns”. (p. 10)
Van den Hoeven and Wandel [53]	“The typology map depicting the vulnerability of inhabitants shows that, in particular, the neighbourhoods in the western part of the city require additional attention to prevent health related risks during severe heat waves. Here, an accumulation of key factors place the elderly and infants more at risk than in other parts of the city due to the lower quality of life of the neighbourhood and the poorer energy efficiency of the buildings”. (p. 87)
Dugord *et al.* 2014 [54]	“We argue that in those areas further soil sealing should be avoided and vegetation density should be increased. In reurbanizing cities such as Berlin, suitable sites for new built-up areas should be identified at an adequate distance from such risk prone areas to control building density”. (p. 97)
Merbitz *et al.* 2012 [55]	“The positive effects of urban green areas and open spaces on air quality and thermal comfort can be clearly deflected from the geo-statistical results”. (p. 105)
Oven *et al.* 2012 [56]	“Our findings therefore suggest that, ideally, risk to built infrastructure supporting older people’s care should be assessed in terms of multiple facets of hazard and vulnerability”. (p. 23)
Keramitsoglou *et al.* 2013 [57]	“This (information) can be useful for targeted prevention measures (short-term planning) or even UHI mitigation planning at city level (long-term planning)”. (p. 8255)
Norton *et al.* 2015	“Despite the increasing amount of research on how Urban Green Infrastructure (UGI) can prevent climatic extremes in urban areas, our understanding remains fragmented and the level of ‘take up’ by urban planners is low. We have presented, justified and applied a hierarchical decision framework that prioritises high risk neighbourhoods and then selects the most appropriate UGI elements for various contexts. Much work remains to be done, especially in determining the optimal arrangement of UGI in a street canyon or the wider urban landscape but there is sufficient information available for local governing bodies to take positive, preventive action and start mitigating high urban temperatures using UGI”. (p. 136)

**Table A2:** Policy recommendations “*a-priori*”/inductive heat vulnerability mapping using health data to test the index.

Reference	Policy Recommendation
Reid *et al.* 2012 [21]	“Results suggest that the HVI can be used to identify areas with increased risks of adverse health outcomes in general, and that it may identify areas at increased risk of heat-related illness and possibly other heat-related outcomes on abnormally hot days. (…) Targeting resources toward decreasing inequities in vulnerability now may increase communities’ resilience to multiple hazards to health in the future”. (p. 719)
Harlan *et al.* 2013 [23]	“Place-based indicators of vulnerability are complements and not substitutes for person-level risk variables. Surface temperature might be used as a single indicator in Maricopa County to identify the most heat-vulnerable neighborhoods. However, more attention to the socioecological complexities of climate mitigation and adaptation is a high public health priority”. (p. 202)
Wolf *et al.* 2013b [24]	“That the performance of a relatively complex multivariate index and a single variable index of heat vulnerability appear to be health outcome dependent raises the question as to whether index parsimony is indeed more important than credibility in a verification and ultimately an application/ decision making context”. (p. 44)
Maier *et al.* 2014 [22]	“This study demonstrates that the modified HVI can be applied outside of metropolitan areas in a southern state and can accurately identify vulnerable populations based on health outcome data. (…) By extending the HVI across the state, public safety officials may be able to target the most vulnerable populations in an attempt to save lives during dangerously hot conditions”. (p. 261)
Chuang *et al.* 2015 [27]	The overall likelihood ratio test shows that factors 1 (socioeconomic deprivation) and 3 (social isolation) are statistically significant predictors of heat hospitalization. Suggestions: Relocation of resources to neighborhoods with high HVI scores; opening cooling centers, providing information about how to prevent heat-related illness to disadvantaged populations, and increasing the efficiency and affordability of residential AC and ventilation, programs to prevent diabetes and to care for people living alone.
Crider 2014 [37]	Mapping allows to identify areas of greater risk from factors like occupation and obesity, singly or in combination and to plan accordingly”. (p. 20)
Houghton *et al.* 2012 [59]	This project confirmed that the platform Geospatial Emergency Management Support System (GEMSS) has the potential to support multiple goals including (a) ongoing monitoring and visualization; (b) providing open-source tool for policy action impacts; (c) tracking status of climate change policies; (d) raising awareness; and (e) providing a basis for epidemiologic research. (p. 43)

**Table A3:** Policy recommendations on mapping health outcomes and exploring vulnerability factors.

Reference	Policy Recommendation
Uejio *et al.* 2011 [38]	“There is a need to expand heat emergency plans that identify at-risk populations domestically and abroad. Mapping heat distress or mortality risk highlights important health inequalities and can be used to target educational or public health interventions”. (p. 505)
Johnson *et al.* 2012 [39]	“Similar analysis could be used to support decision processes before a municipal heat wave or during the disaster itself to benefit mitigation”. (p. 29)
Hondula *et al.* 2012 [41]	“In the case of alerting the public, localities associated with excess mortality could receive additional notification or special forecasts when hot conditions are expected. These places are also prime candidates for facilities that can help residents escape the impact of high apparent temperatures”. (p. 10)
Boumans *et al.* 2014 [43]	“This pilot model demonstrated a dynamic spatial model structure for providing this type of information for a particular geographic location and set of health outcomes of concern. Further model development will be directed toward application to other geographic locations and expanding the set of health outcomes and environment–health interactions”. (p. 98)
Heaton *et al.* 2014 [44]	“While this study was useful in identifying environmental and socio-demographic factors of vulnerability, a future analysis would be to look more closely at each block group to determine why a block is vulnerable to heat or not. That is, individually comparing block groups will lead to a better understanding of the differential vulnerability....” (p. 32)
Loughnan *et al.* 2012 [40]	“The spatial vulnerability index developed in this study provides critical information for policy makers and planners, healthcare professionals, and ancillary services. Each of the local government areas in Melbourne can now identify POA in its jurisdictions that are most at risk. Areas of increased risk within each POA can be identified using local knowledge or by reexamination of the data at a census collection district level. Such information can then be used to direct services such as community education, emergency management, heat-health adaptation strategies, and direct short-term and longer-term redevelopment and refurbishment of existing dwellings to mitigate the effects of heat in urban areas”. (p. 10)
Klein Rosenthal *et al.* 2014 [42]	In addition to low income and lack of air conditioning, also neighborhood stability, economic hardship, and building conditions in New York City neighborhoods need to be reflected in planning and design of strategies of urban heat island mitigation. Measures can include provision of access to cooling for seniors during extreme hot weather and policies to improve the housing conditions of elderly residents.
Johnson and Wilson 2012 [62]	“Maps depicting spatial variation of risk within a city would allow health professionals to concentrate intervention strategies in the areas identified as high risk. This could involve the formation of community volunteers to check in on elderly individuals in the highest risk areas during EHEs. Additionally, these areas of high risk could serve to focus on the distribution of resources, such as emergency clinics and cooling stations, which are common public health intervention strategies implemented during EHEs”. (p. 430)
Hondula 2014 [45]	Areas with higher percentages of high-income earners were at less risk and areas with higher population density were at higher risk. In 16 (out of 158) districts with significant relationships between heat and hospital admission, targeted efforts could be envisaged.
Schuster *et al.* 2014 [63]	“We argue that temporal aggregation could be a powerful option for studying heat mortality even when daily data are available, since it allows for the investigation of spatial mortality variation at a much finer scale”. (p. 145)
Kovach *et al.* 2015 [64]	“Ultimately, results from the present study highlight locations where targeted public health interventions, future research, and resource allocation can mitigate emergency department admissions from heat-related illness”. (p. 182)
Hattis *et al.* 2012 [65]	“These results suggest that, at least in Massachusetts, an area’s demographics may be more important to its heat-related mortality than its level of urbaniza- tion, at least as captured by the specific variables used in this study. (...) Further research is needed to determine the factors that affect heat- related mortality in rural areas, especially in light of expected temperature increases”. (p. 51)
